# Study of late toxicity biomarkers of locally advanced head and neck cancer patients treated with radiotherapy plus cisplatin or cetuximab points to the relevance of skin macrophages (TOX-TTCC-2015-01)

**DOI:** 10.1007/s12094-024-03526-0

**Published:** 2024-05-23

**Authors:** Antonio Rullan, Juan A. Marín-Jiménez, Alicia Lozano, Oriol Bermejo, Lorena Arribas, Nuria Ruiz, Isabel Linares, Miren Taberna, Xavi Pérez, María Plana, Marc Oliva, Ricard Mesía

**Affiliations:** 1 Department of Medical Oncology, Catalan Institute of Oncology (ICO), L’Hospitalet de Llobregat, Barcelona, Spain; 2 Department of Radiation Oncology, Catalan Institute of Oncology (ICO), L’Hospitalet de Llobregat, Barcelona, Spain; 3 Departament of Plastic and Reconstructive Surgery, Bellvitge University Hospital, L’Hospitalet de Llobregat, Barcelona, Spain; 4 Clinical Nutrition Unit, Catalan Institute of Oncology (ICO), L’Hospitalet de Llobregat, Barcelona, Spain; 5 Department of Pathology, Bellvitge University Hospital, L’Hospitalet de Llobregat, Barcelona, Spain; 6 Unit of Human Anatomy and Embryology, Department of Pathology and Experimental Therapeutics, Faculty of Medicine and Health Sciences (Bellvitge Campus), University of Barcelona, Barcelona, Spain; 7 Clinical Investigation Unit (UIC), Catalan Institute of Oncology (ICO) L’Hospitalet, Barcelona, Spain; 8 Department of Medical Oncology, Catalan Institute of Oncology (ICO), B-ARGO Group, IGTP, Badalona, Barcelona, Spain

**Keywords:** Head and neck cancer, Radiotherapy, Chemotherapy, Cetuximab, Cisplatin, Biomarkers, Late toxicity, Macrophages

## Abstract

**Purpose:**

Radiotherapy (RT) with concomitant cisplatin (CRT) or cetuximab (ERT) are accepted treatment options for locally advanced squamous cell carcinoma of the head and neck (LA-SCCHN). Long-term adverse events (AEs) have a vast impact on patients’ quality of life. This study explored tissue biomarkers which could help predict late toxicity.

**Methods/patients:**

Single-institution prospective study including patients aged ≥ 18 with histologically confirmed newly diagnosed LA-SCCHN treated with RT and either concomitant cisplatin q3w or weekly cetuximab, according to institutional protocols. All patients underwent pre- and post-treatment skin biopsies of neck regions included in the clinical target volume. Angiogenesis, macrophages, and extracellular matrix (ECM) markers were evaluated by immunohistochemistry (IHC).

**Results:**

From April 15, 2016, to December 11, 2017; 31 patients were evaluated [CRT = 12 (38.7%) and ERT = 19 (61.3%)]. 27 patients (87%) had received induction chemotherapy. All patients finished RT as planned. IHC expression of vasculature (CD34) and collagen (Masson’s Trichrome) did not differ significantly between and within CRT and ERT arms. Conversely, an increased CD68 and CD163 macrophage infiltration expression was observed after treatment, without significant impact of treatment modality. Patients with higher late toxicity showed lower expression of macrophage markers in pre-treatment samples compared with those with lower late toxicity, with statistically significant differences for CD68.

**Conclusions:**

Angiogenesis and ECM biomarkers did not differ significantly between CRT and ERT. Macrophage markers increased after both treatments and deserve further investigation as predictors of late toxicity in LA-SCCHN patients. [Protocol code: TOX-TTCC-2015-01/Spanish registry of clinical studies (REec): 2015-003012-21/Date of registration: 27/01/2016].

**Supplementary Information:**

The online version contains supplementary material available at 10.1007/s12094-024-03526-0.

## Introduction

Together with primary surgery and adjuvant treatment, concurrent RT and systemic treatment is the standard therapy for LA-SCCHN [1]. It is the first option for patients with non-resectable tumors and for those with resectable disease who are not good surgical candidates [1]. Both cisplatin and cetuximab in combination with RT have demonstrated improved locoregional control and overall survival compared with RT alone [2, 3]. CRT has shown to be superior to ERT in HPV-positive oropharyngeal LA-SCCHN [4, 5]. Although not formally compared, CRT is preferred to ERT for HPV-negative tumors whenever possible [1]. For this reason, eligibility for cisplatin is the leading criterion when choosing between the two treatments, while susceptibility to long-term AEs is often overlooked. However, there is comprehensive evidence of their significant impact on the quality of life of patients, with up to 43% of LA-SCCHN patients reporting severe late toxicity after CRT treatment [6–9].

Several studies have sought to determine the factors associated to late toxicity caused by concurrent therapies for LA-SCCHN patients and have highlighted the need of considering them for treatment selection. Clinical factors—such as age, female sex, primary and nodal tumor burden and tumor location (larynx and hypopharynx) are independent predictors of late treatment toxicity [8]. Pre-treatment nutritional status and weight loss during RT are also associated with the appearance of late-onset AEs [10, 11]. Other predictors of RT-induced toxicity include a prognostic nutritional index that combines serum albumin and lymphocyte count [12], and the quantification of radiation-induced apoptosis (RIA) in peripheral blood lymphocytes [13] which predicted individual sensitivity to RT. In another study, tumor genetic alterations detected by next-generation sequencing in the tumor correlated with toxicity outcomes upon RT [14]. More recently, machine- and deep-learning models have been developed to predict toxicity to treatment for head and neck cancer patients [15, 16].

All these studies remark on the impact of RT and multimodality treatment on quality of life of head and neck cancer patients, even many years after treatment completion. In view of that, there is an urgent need to discover biomarkers associated with severe late toxicity. In this study, we sought to determine the effects of CRT and ERT in healthy tissue from patients with LA-SCCHN. We evaluated the changes in microvasculature, ECM and macrophage infiltration and their correlation with late toxicity to both treatments.

## Materials and methods

### Study design and patients

This is a single center, non-randomized, prospective study, focused on tissue biomarker analysis. Opportunistic recruitment was performed sequentially in the Head and Neck Multidisciplinary Unit for patients that were candidates to radical RT in combination with platinum-based chemotherapy or cetuximab. Previous induction chemotherapy was accepted. Patients were > 18 years of age, able to provide written informed consent, and had a histologically confirmed LA-SCCHN. Patients with medical conditions that increased the risk of complications derived from a skin biopsy (e.g., severe skin diseases) were excluded.

### Treatment and procedures

Concurrent RT with curative intent was administered to all patients in the study. All participants were treated with 6 Megavolt X-ray beam, volumetric arc radiation therapy (VMAT) at 70 Gy for tumor and involved nodes and 54.12 Gy for non-involved nodes, scheduled at 2.12 Gy/dose with one dose/day, 5 days/week. Patients treated with CRT received cisplatin 100 mg/m^2^, days 1,22 and 43. In those treated with ERT cetuximab was initiated with a 400 mg/m^2^ loading dose 1 week before RT followed by weekly cetuximab at 250 mg/m^2^ for the duration of RT. Patients treatment was decided at the multidisciplinary tumor board according to institutional protocols and local guidelines [17]. According to these recommendations, those patients with unresectable disease or who were candidates for organ preservation protocols (larynx/hypopharynx) were treated with induction chemotherapy with the standard docetaxel, cisplatin, and fluorouracil (TPF) regimen.

As part of the trial, patients were subjected to a 4 mm cutaneous punch biopsy under local anesthesia prior to treatment. The biopsy was performed in healthy skin from cervical node level II, homolateral to the site of primary tumor, as it was expected that the area would be irradiated in all cases with low radiation dose variability. Two months after completing RT, a subsequent biopsy was taken from the area of the neck corresponding to the planned clinical target volume. Acute toxicity was evaluated using the Common Terminology Criteria for Adverse Events v4.0. Late toxicity was defined as per RTOG/EORTC late radiation morbidity scoring system and collected 1 year after the end of RT. Nutritional status of the patients was evaluated by the Clinical Nutrition Unit before concurrent treatment and 2, 6 and 12 months after RT completion. Nutritional assessment was performed using the Patient-Generated Subjective Global Assessment (PG-SGA) [18]. For the biomarker analysis, patients were classified according to reported late toxicity in toxicity high (Tox^High^: > 2 AEs and/or any ≥ grade 2) and toxicity low (Tox^Low^: < 2 AEs and grade 1).

### Immunohistochemistry

Biomarker analysis was performed by IHC staining on the FFPE slides from paired biopsies from each patient. Both pre- and post-treatment samples were assessed for: (a) the presence of endothelial and lymphatic vessels with CD34 as vasculature marker, (b) collagen deposition in the ECM through the Masson’s trichrome staining, and (c) infiltration of pro- and anti-inflammatory macrophages with CD68 and CD163, respectively. CD34, CD68 and CD163 were scored as a percentage of stained cells (IHC positive stained cells/total number cells × 100), while for Masson’s trichrome, the percentage of the stained area was calculated.

Skin biopsies were fixed using neutral buffered formalin for 24 h before embedding in paraffin. Samples were baked and dewaxed before staining with 3, 3’-diaminobenzidine-based IHC according to standard procedures in a Ventana autostainer. The antibodies used were: CD34 (clone QBEnd10, Agilent-Dako #GA362). CD68 (clone KP-1, Ventana #790-2931), CD163 (clone 10D6, Biocare Medical #CM353AK). Masson’s Trichrome stain was performed with the Agilent Dako Stain Kit #AR173 according to the manufacturer’s instructions. The slides were scanned at 20 × using a Roche-Ventana DP 200 slide scanner. The analysis was performed using QuPath 0.3.0 [19]. The relevant subcutaneous areas were selected manually. After optimization, we run the “cell detection” and the “positive cell detection” plugin from Qupath to calculate the percentage of positive cells.

### Statistical analysis

Statistical analysis was performed using Statistical Package for the Social Sciences (SPSS) v23. Graphs and statistics for the biomarker analysis were performed using GraphPad Prism v9.0. Chi-square (for trend) and Fisher’s exact tests were used for differences between proportions. Paired and unpaired Welch’s t test were used for mean comparisons. A mixed-effect model was adjusted for the analysis across different treatment and timepoints. All hypothesis tests are two-tailed. The significance level for all the statistical tests was set to 0.05.

## Results

### Patient and tumor characteristics

**Fig. 1:**
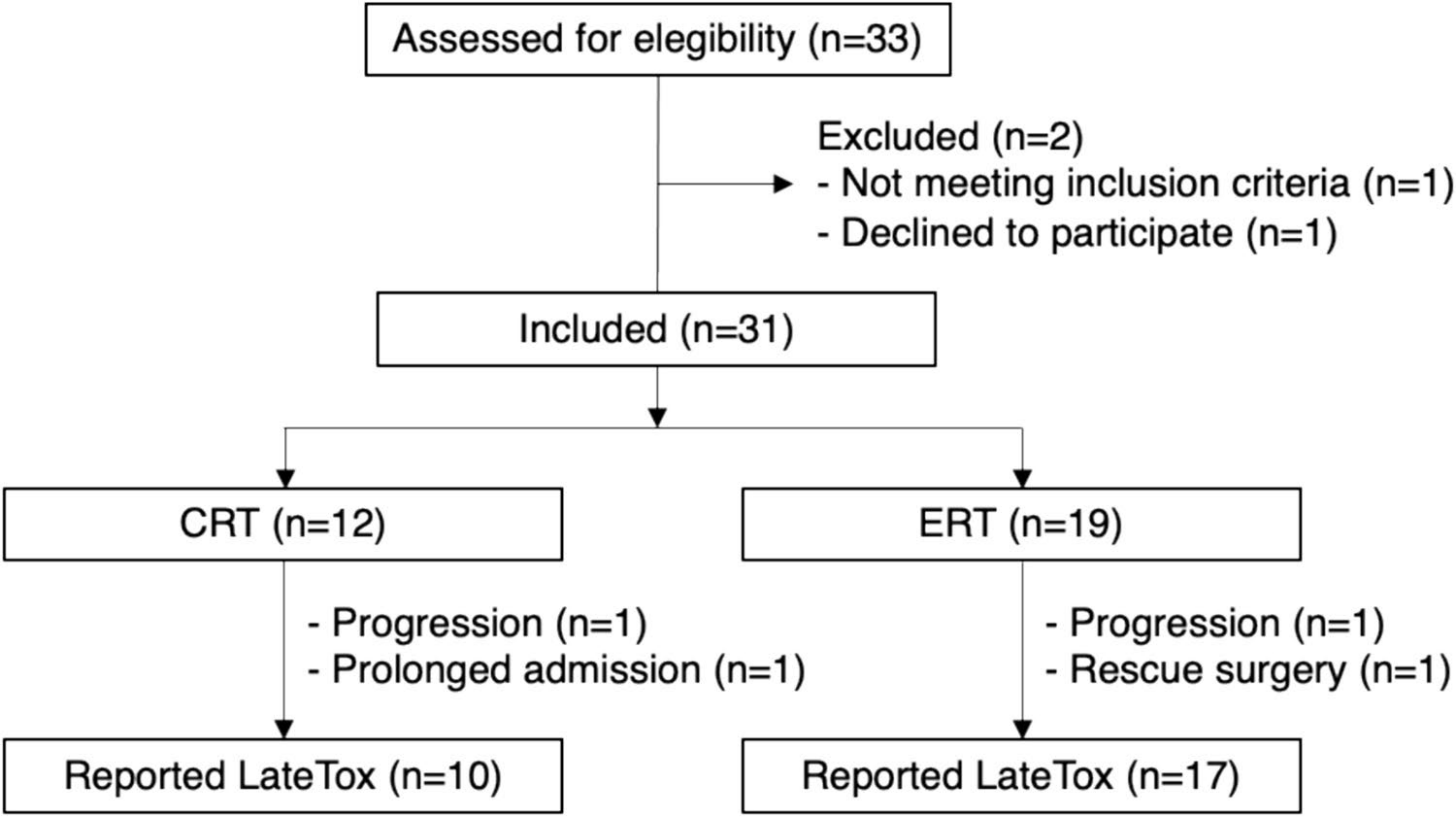
Flow diagram of the study

Between April 2016 and December 2017, 33 patients were assessed for eligibility (Fig. 1). Of them, 31 patients met all the eligibility criteria and were included in the study: 12 patients received CRT and 19 patients received ERT. Baseline characteristics by treatment group are summarized in Table 1. Most of the patients were male and median age was 62 (range 44–76). Baseline characteristics and pre-treatment nutritional status were well balanced across treatment groups. With n = 9 each, hypopharynx, oropharynx, and larynx were the most represented primary tumor locations. Although the differences were not significant, CRT treated patients showed a higher tumor (T) burden compared with ERT patients (T4 = 64% vs 33%, P value = 0.14).

**Table 1:** Patient and tumor baseline characteristics according to treatment groups

	CRT (N = 12)	ERT (N = 19)	*P value*
Age (years)			
*Median (range)*	60.5 (45–69)	63 (44–76)	*0.84*
Gender—*n (%)*			*0.54*
Male	10 (83)	18 (95)	
Female	2 (17)	1 (5)	
ECOG PS—*n (%)*			*0.99*
0	0	1 (5)	
1	12 (100)	18 (95)	
Tobacco*—n (%)*			0.27*
Non-smoker	0	1 (5)	
Former smoker	3 (25)	8 (42)	
Active smoker	9 (75)	10 (53)	
Alcohol—*n (%)*			0.99*
Non-alcohol use	1 (8)	3 (16)	
Former alcohol use	4 (33)	6 (32)	
Active user	7 (58)	10 (52)	
Pre-treatment weight (Kg)			
*Mean (standard deviation)*	71.1 (12.8)	73.7 (20.0)	*0.68*
Pre-treatment PG-SGA*—n (%)*			0.12*
A, well-nourished	4 (33)	11 (58)	
B, moderate/suspected malnutrition	5 (42)	2 (11)	
C, severly malnourished	1 (8)	2 (11)	
Unknown	2 (17)	4 (21)	
Tumor location—*n (%)*			*0.92*
Oral cavity	1 (8)	2 (11)	
Oropharynx	3 (25)	6 (32)	
Larynx	3 (25)	6 (32)	
Hypopharynx	4 (33)	5 (26)	
Unknown primary location	1 (8)	0	
Primary tumor (TNM 7th ed.)—*n (%)*			0.14*
Tx	1 (8)	1 (5)	
T1	0	1 (5)	
T2	1 (8)	1 (5)	
T3	3 (25)	10 (53)	
T4	7 (58)	6 (32)	
Lymph nodes (TNM 7th ed.)—*n (%)*			0.70*
N0	4 (33)	8 (42)	
N1	1 (8)	0	
N2a	0	1 (5)	
N2b	3 (25)	5 (26)	
N2c	2 (17)	2 (11)	
N3	2 (17)	3 (16)	
RT volumes (cm^3^)*—median (range)*			
Tumor (T)	42 (3–153)	47 (6–118)	*0.73*
Lymph nodes (N)	300 (221–400)	317 (175–533)	*0.17*

*PG-SGA* Patient-Generated Subjective Global Assessment (PG-SGA)

*Grouped comparisons: Tobacco and alcohol, non/former vs active; PG-SGA, A vs B/C; Primary tumor, T ≤ 3 vs T > 3; Lymph nodes, N ≤ 2b vs N > 2b

### Treatment and patients’ outcomes

All the patients included in the study completed RT as planned. Total irradiated tumor and lymph node volumes were similar by treatment groups (Table 1). Volumetric data on parapharyngeal, lymph node regions, parotid glands, oral cavity, and larynx are detailed in Supplementary Table 1. Due to local practice by the time of the study, most patients (n = 27, 87.1%) had received prior TPF induction chemotherapy. Two patients per group underwent exclusive concurrent treatment. Of those patients treated with CRT, 11 (92%) received at least one dose of cisplatin, and 8 (72.7%) completed 3 cycles of cisplatin. Patient #8 was administered three cycles of carboplatin due to renal function impairment and patient #12 switched to carboplatin after 1 cycle of cisplatin due to grade 2 peripheral neuropathy. 14 patients (73.7%) treated with ERT received weekly cetuximab until RT completion (at least 8 doses). Details of treatment compliance are summarized in Supplementary Table 2.

Tumor evaluation with neck CT scan was performed 2 months after last RT dose. 24 patients (77%) achieved a complete response after concurrent treatment. Three (25%) and 4 (21.1%) patients in the CRT and ERT treatment arms respectively underwent lymph node dissection due to persistent node disease. With data cut-off on May 30, 2020, three (25%) CRT and 3 (15.8%) ERT treated patients showed disease recurrence. Four patients died during the follow-up out of 19 (21.1%) treated with ERT, due to cancer progression (n = 3) and non-cancer related heart failure (n = 1). None of the CRT treated patients were dead by data cut-off.

### Toxicity

#### Acute toxicity

Adverse events (AEs) were monitored during the concurrent treatment as per local protocol. Acute toxicity to CRT or ERT was recorded according to CTCAE v4.0. Acute adverse events are detailed in Supplementary Table 3. All patients had G1-2 acute toxicity to CRT/ERT. 18 patients (58%) experienced G3-4 acute adverse events to concurrent treatment. Dysgeusia (25%), oral mucositis (16.7%) and xerostomia (16.7%) were the most prevalent G3 AEs to CRT. One patient experienced G4 neutropenia to CRT. Grade 3 oral mucositis (52.6%), dysphagia (10.5%) and dermatitis (10.5%) were reported during ERT. One patient was diagnosed with G4 pneumonia while on ERT treatment, requiring ICU admission. Complications derived from the skin biopsy were not observed.

In total, 5 patients (16%) discontinued treatment due to acute toxicity. Three patients (15.8%) permanently discontinued cetuximab due to G3 oral mucositis, extensive and permanent G3 skin rash and G4 pneumonia, respectively. Two patients (16.7%) omitted the third administration of cisplatin due G3 and G4 neutropenia. There were no toxic deaths during the study treatment. Of note, 2 (16.7%) versus 8 (42.1%) patients needed a nasogastric feeding tube during CRT and ERT treatment, respectively.

#### Late toxicity

Due to death (n = 1) and disease progression (n = 3), late toxicity evaluation was not possible in two patients per group. Late toxicity by treatment group is detailed in Table 2. G1-2 xerostomia (80%), peripheral neuropathy (80%) and fatigue (30%) were the most prevalent late AEs after CRT. Among ERT treated patients, G1-2 xerostomia (64.7%), fatigue (47.1%), dysphagia (17.6%) and peripheral neuropathy (17.6%) were present 1 year after treatment. One patient experienced G4 osteonecrosis after CRT treatment. One patient in the ERT group still needed tracheostomy by the time of late toxicity evaluation.

**Table 2:** Late toxicity according to treatment groups

	CRT (N = 10)		ERT (N = 17)	
AE	Grade	N (%)	Grade	N (%)
Xerostomia	1	7 (70)	1	10 (58.8)
	2	1 (10)	2	1 (5.9)
Larynx dysfunction	1	1 (10)	–	0
Oral mucositis	1	1 (10)	–	0
Osteonecrosis	4	1 (10)	–	0
Anorexia	–	0	1	1 (5.9)
Asthenia	1	2 (20)	1	7 (41.2)
	2	1 (10)	2	1 (5.9)
Dysphagia	1	2 (20)	1	3 (17.6)
			3	3 (17.6)
Dysphonia	1	2 (20)	1	1 (5.9)
Peripheral neuropathy	1	6 (60)	1	3 (17.6)
	2	2 (20)		
Radiodermitis	–	0	1	2 (11.8)
Skin rash	–	0	1	2 (11.8)
Trismus	–	0	1	1 (5.9)

### Biomarker analysis

#### Concurrent treatment did not alter vascular density or collagen deposition

**Fig. 2:**
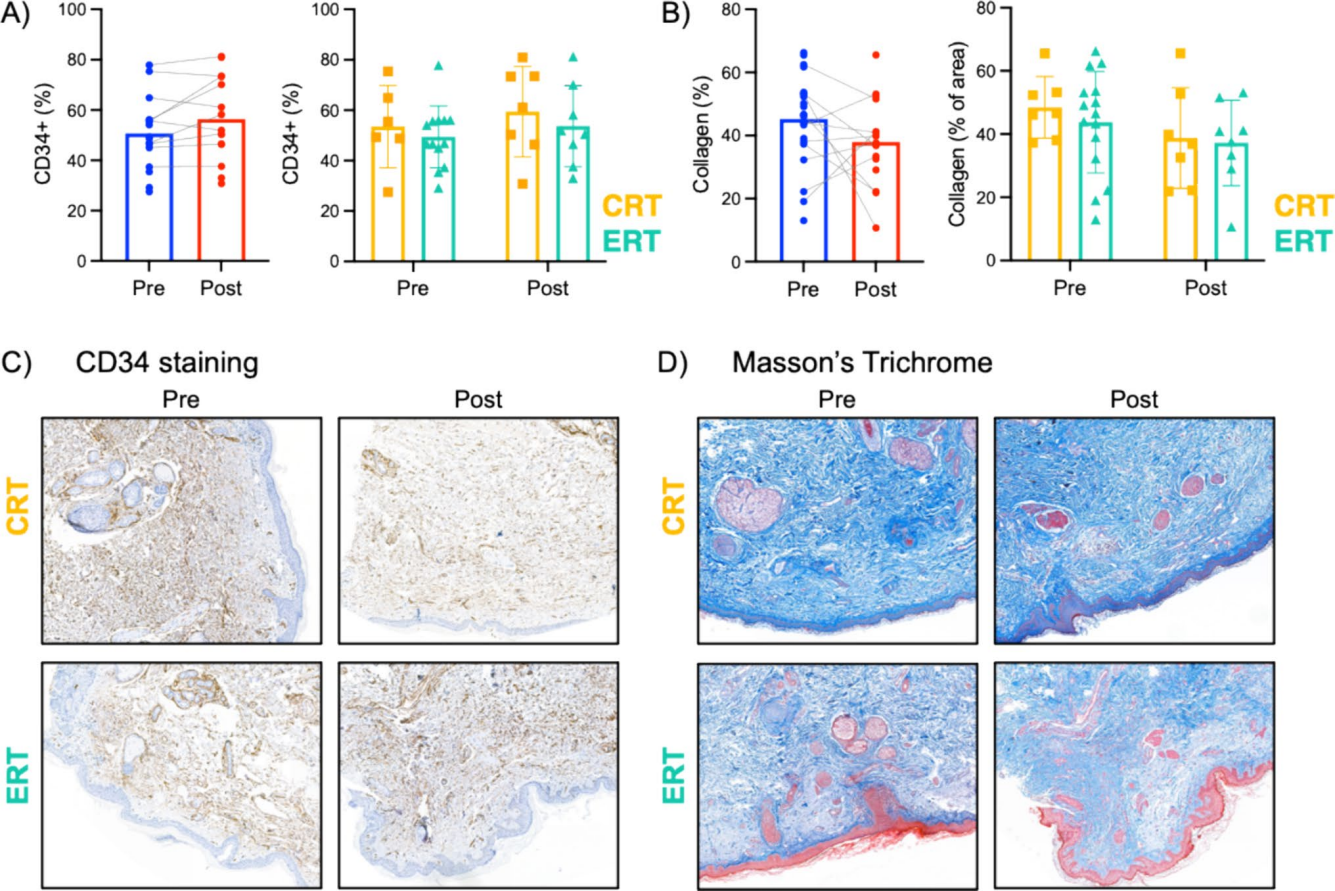
**A** CD34 expression before (Pre, n = 19) and 2 months after treatment (Post, n = 15) and by treatment group. **B** Collagen deposition before (n = 22) and 2 months after treatment (n = 15) and by treatment group. **C** Illustrative images from CD34 IHC. **D** Illustrative images from Masson’s trichrome staining

For the whole cohort, the mean number of CD34 + stained vascular cells were similar between pre- and post- treatment samples (50.7% vs 56.4%, paired T test P-value = 0.108). There were no significant differences in the percentage of CD34 + cells pre- and post-treatment between CRT (53.5% vs 59.5%) and ERT (49.5% vs 53.7%) treated patients (P-values: time = 0.092, treatment = 0.598) (Fig. 2A). Similarly, collagen density was similar before and after concurrent treatment (45.3% vs 38.0%, paired-T test P = 0.332) and by treatment group: CRT (48.5% vs 38.8%,) and ERT (43.7% vs 37.2%, P-values: time = 0.135, treatment = 0.482) (Fig. 2B). Illustrative images of CD34 and Masson’s staining are shown in Fig. 2C, D, respectively.

#### Macrophage infiltration increases after concurrent treatment with either CRT or ERT

**Fig. 3:**
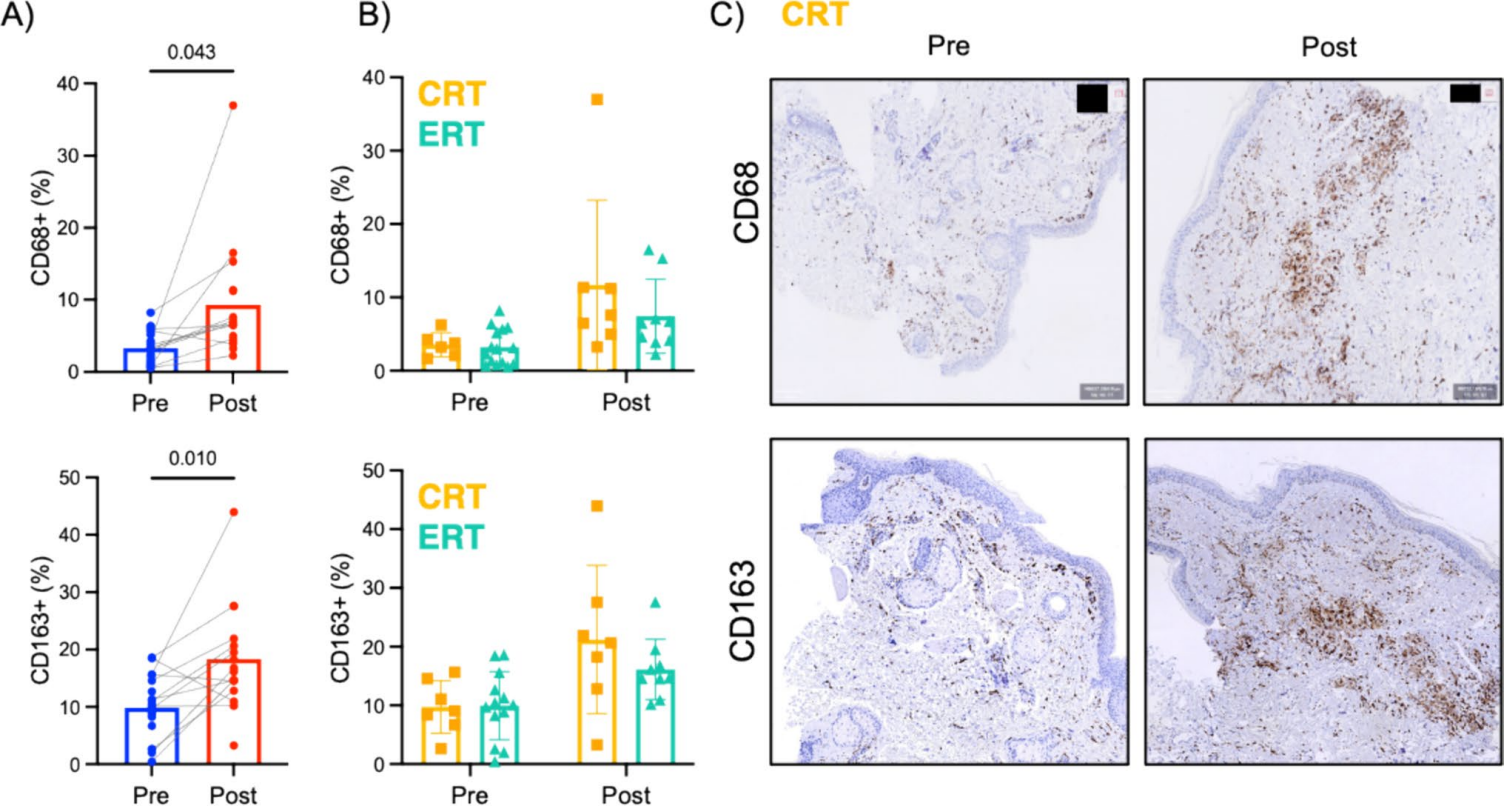
**A** CD68 expression before (Pre, n = 21) and 2 months after treatment (Post, n = 16) and CD163 expression before (Pre, n = 20) and 2 months after treatment (Post, n = 16). **B** CD68 and CD163 expression by treatment groups. **C** Illustrative examples with significant increase in CD68 + and CD163 + macrophages after CRT treatment in patient #29

For the analysis of macrophage infiltration, CD68 and CD163 markers were used to identify likely pro-inflammatory and anti-inflammatory macrophages, respectively. Overall, we observed an increased macrophage infiltration after concurrent treatment. The percentage of macrophage infiltration (MΦ) increased significantly after treatment for both CD68 + MΦ (3.3–9.3%, Paired-T test P = 0.043) and CD163 + MΦ (9.9–18.3%, Paired-T test P = 0.010) (Fig. 3A). The increase in CD68 + MΦ was observed after concurrent treatment regardless treatment modality (CRT: 3.5% vs 11.7%, ERT: 3.2% vs 7.4%; P-values: time = 0.014, treatment = 0.301) (Fig. 3B). A similar increase during treatment was observed for CD163 + MΦ in CRT (9.7% vs 21.2%) and ERT (9.9% vs 16.1%) treated patients (P-values: time = 0.003, treatment = 0.484) (Fig. 3B). Of note, one patient treated with cisplatin (patient #29) showed a remarkable increase in CD163 + MΦ after treatment (15.6% vs 44%) (Fig. 3C).

#### Analysis by late toxicity groups

**Fig. 4:**
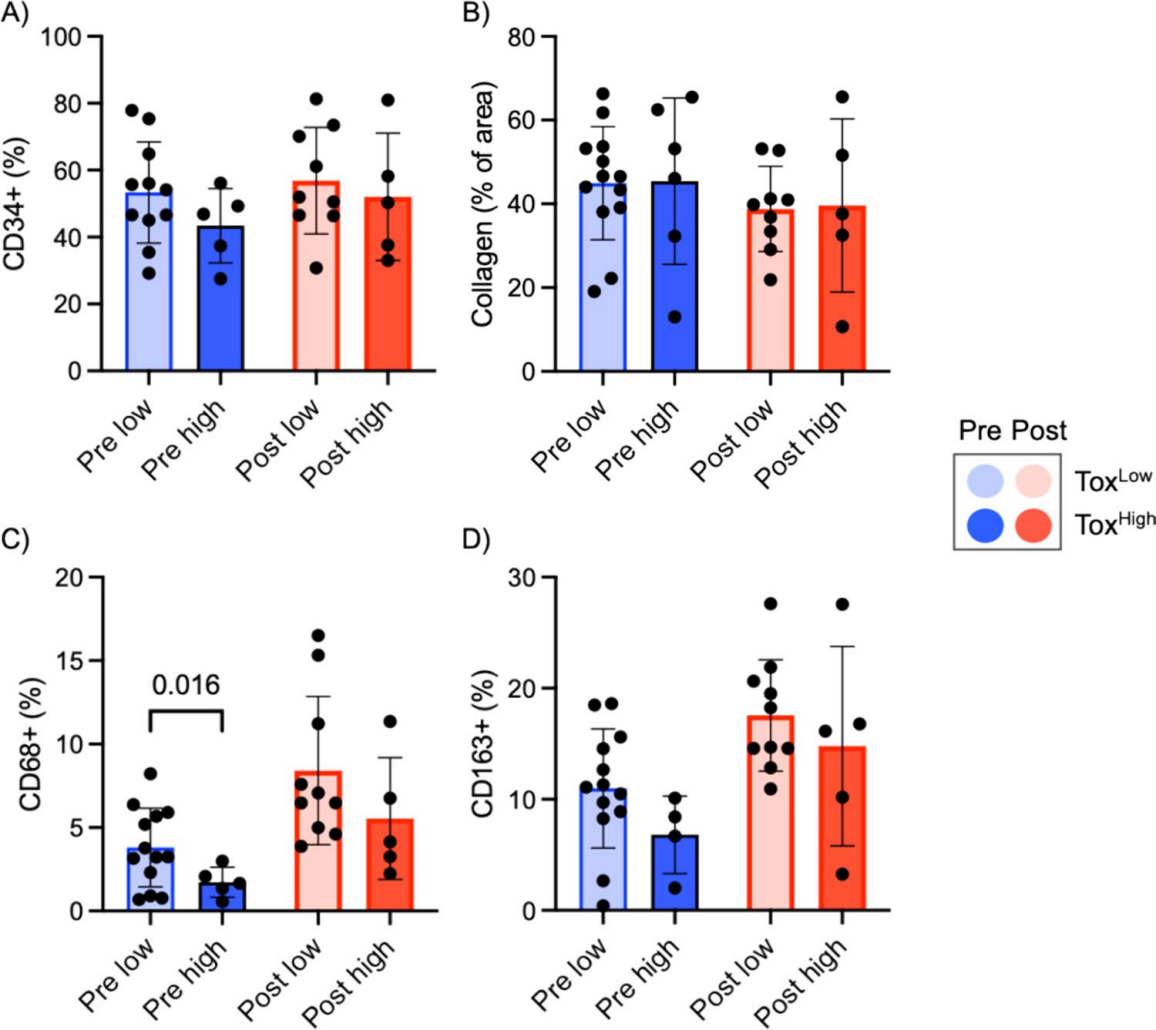
Biomarker analyses across treatment among late toxicity groups were performed for CD34 (**A**), Masson’s trichrome (**B**), CD68 (**C**) and CD163 (**D**). Unpaired t test with Welch’s correction were used to compare pre-treatment levels of macrophage infiltration

According to the number and grade of late toxicity AEs, those patients with reported late toxicity (n = 27), were classified as late toxicity low (Tox^low^: < 2 AEs and grade 1, n = 18 (66%)) and high (Tox^high^: > 2 AEs and/or any ≥ grade 2, n = 9 (33%)) patients. Tox^high^ patients were more abundant among CRT treated patients (4/10, 40%) compared with ERT treated patients (5/17, 29%). Vascular structures and collagen deposition were comparable before and after treatment for Tox^low^ and Tox^high^ patients and seemed not to change during concurrent treatment (P-values for toxicity and time not significant) (Fig. 4A, B). CD68 + MΦ increased after treatment in both groups regardless of toxicity: Tox^low^ (3.8% vs 8.4%) and Tox^high^ (1.7% vs 5.5%); (P-values for toxicity = 0.062 and time = 0.003) (Fig. 4C). Similarly, CD163 + MΦ also increased after treatment but not differentially across toxicity groups: Tox^low^ (11.0% vs 17.5%) and Tox^high^ (6.8% vs 14.8%); (P-values for toxicity = 0.185 and time = 0.004) (Fig. 4D). Patients within Tox^high^ group showed less macrophage infiltration pre-treatment compared with Tox^low^ patients, although statistical significance was only reached for CD68 + MΦ (P value = 0.016) (Fig. 4C).

### Nutritional evaluation

**Fig. 5:**
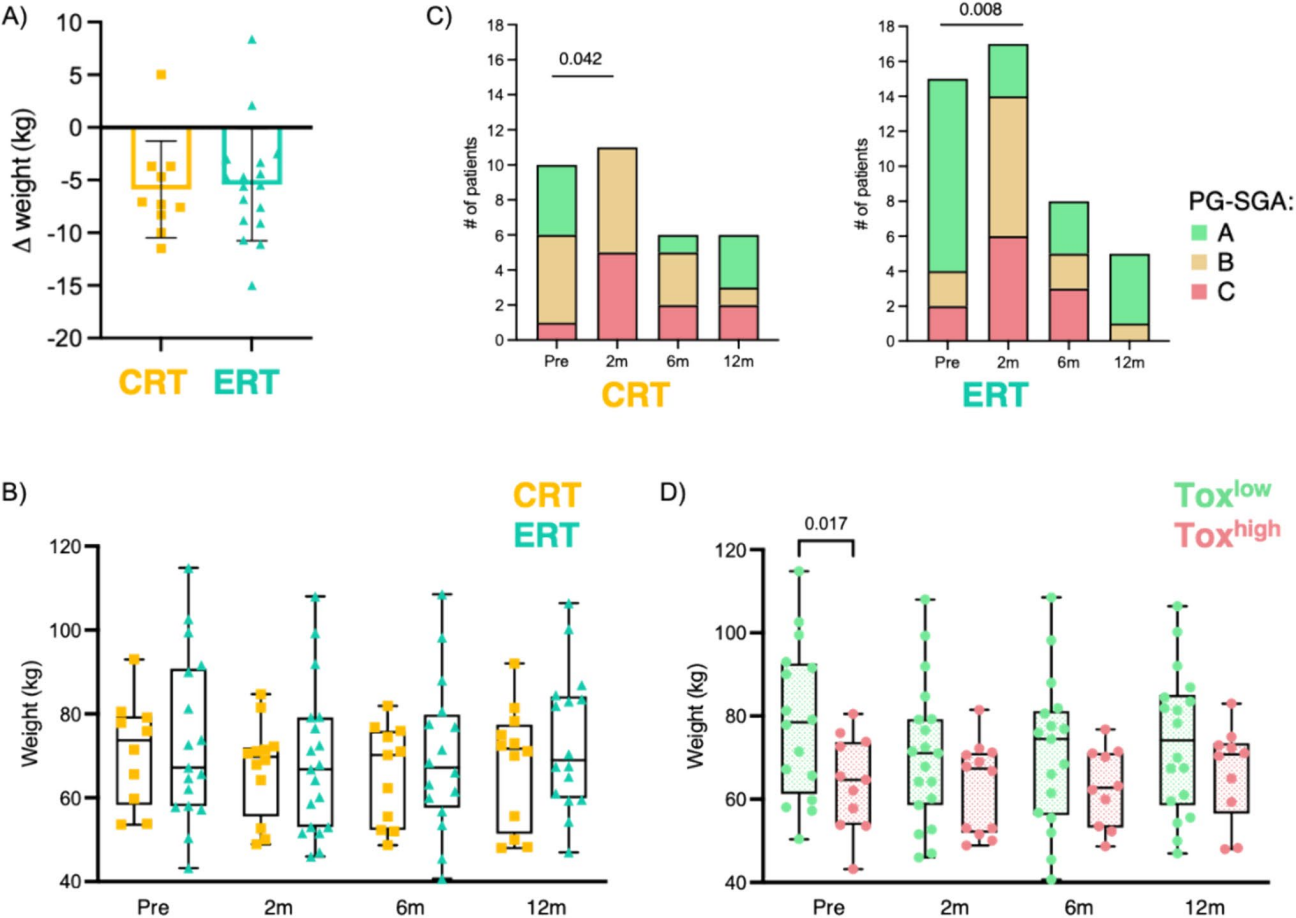
Weight and nutritional evaluation during concurrent treatment and follow-up. **A** Weight variation between baseline and 2 months after RT completion; **B** weight measurement by treatment group; **C** PG-SGA by treatment groups; **D** weight measurement by late toxicity groups

Despite receiving oral nutritional supplementation prior to treatment initiation, most patients experienced weight loss during concurrent treatment, with no statistically significant variances observed between the CRT and ERT cohorts (mean weight change:  – 5.89 kg vs  – 5.44 kg, P value = 0.817) (Fig. 5A). Patients maintained stable weight during the first year post-RT (Fig. 5B). However, the PG-SGA nutritional scores deteriorated in both treatment groups compared to their baseline scores (CRT: p-value = 0.042, ERT: p-value = 0.008) (Fig. 5C). Consequently, enteral nutrition was required for 3 (25%) CRT-treated and 6 (32%) ERT-treated patients. One CRT patient and 3 ERT patients still needed gastrostomy 1 year after RT completion. Of note, the patients who exhibited significant chronic toxicity (Tox^high^) had a lower mean body weight before starting concurrent treatment (Tox^high^ 68.2 vs Tox^low^ 73.8 kg, P value = 0.048) (Fig. 5D).

## Discussion

Late toxicity resulting from multimodality treatment for SCCHN patients can have a significant impact on their quality of life. Currently, there are no predictive biomarkers for late toxicity in patients treated with CRT nor ERT. The aim of this prospective trial was to study healthy skin at baseline and 3 months after RT to generate hypotheses regarding changes in inflammatory and fibrotic components. To our knowledge, this is the first study to prospectively evaluate tissue biomarkers as predictors of late toxicity to concurrent treatment for LA-SCCHN.

Baseline characteristics of our patient cohort were overall representative of the usual population diagnosed with LA SCCHN and treated with CRT and ERT. However, although it is not recommended outside larynx-preservation protocols [1], in our study a high percentage of patients received induction chemotherapy due to the advanced disease stage at diagnosis and local practice at the time of the study. Early and late AEs observed were in line with previously described acute toxicity in the literature and was comparable across the two groups [4, 5]. Given the influence of the nutritional status in late toxicity we collected information about the baseline status and weight loss in both treatment groups. There were no significant differences between patients treated with CRT and ERT. In concordance with the literature, patients that developed higher late toxicity tended to have a lower weight at baseline [10, 11].

Long-term fibrosis resulting from increased deposition of collagen is a hallmark of RT toxicity [20] and has been shown to increase in the skin of irradiated patients [21]. Therefore, we investigated whether changes in collagen density could be detected 2 months after RT. As shown in Fig. 3, there was no significant increase in collagen density in pre- and post-treatment samples for either of the treatment groups. RT induces changes in the microvasculature that can later result in alterations of vascularization and tissue dysfunction [22]. However, we did not observe any changes in CD34 expression in our cohort. We did not find any correlation between toxicity and collagen or CD34 density in our patients.

An explanation for the absence of significant changes in collagen and CD34 is the chosen timepoint, at which there may not yet be evidence of later processes linked to RT toxicity and organ dysfunction. Prior to myofibroblast activation and changes in the deposition of extracellular matrix, there is an inflammation phase linked to tissue injury with agents such as RT and chemotherapy [21]. This inflammatory phase is characterized by increased infiltration of myeloid cells such as macrophages. Indeed, our analysis showed an increase in both pro-inflammatory (CD68 +) and anti-inflammatory (CD163 +) macrophages after treatment with either CRT or ERT, with no significant difference between the two treatment groups.

Interestingly, we observed a trend in which patients with higher late toxicity had a lower macrophage infiltrate at baseline. One hypothesis is that a reduced macrophage presence can result in deficient clearance of damaged cells after RT, leading to a more accentuated chronic inflammation and late toxicity. However, the limited sample size, particularly in the high toxicity group, does not allow us to draw definitive conclusions. Another limitation of our trial is the fact that most of our patients received induction chemotherapy, which may have influenced the cellular behavior in the skin prior to radiotherapy.

## Conclusions

Our study demonstrates the feasibility of analyzing the effect of RT and systemic treatments on healthy skin. Further studies are needed to determine the role of macrophages in toxicity outcomes for these patients. Discovering a biomarker that accurately predicts severe toxicity risks for LA-SCCHN patients would be extremely helpful for clinical decision making in this setting.

## Supplementary Information

Below is the link to the electronic supplementary material.

Supplementary file1 (DOCX 17 KB)

## Data Availability

The data that support the findings of this study are available from the corresponding author, RM, upon reasonable request.
