# Supplementary material for: Study of late toxicity biomarkers of locally advanced head and neck cancer patients treated with radiotherapy plus cisplatin or cetuximab points to the relevance of skin macrophages (TOX-TTCC-2015-01)
Source: Clin Transl Oncol. 2024 May 23;26(12):3003–12. doi: 10.1007/s12094-024-03526-0 (PMC11564235; doi:10.1007/s12094-024-03526-0)
Supplement: Supplementary file 1 — Supplementary file1 (DOCX 17 KB) [file 12094_2024_3526_MOESM1_ESM.docx]

**Supplementary Table 1**. Number of patients receiving radiation to parapharyngeal, ganglionar (IB bilateral, III, IV, and V), parotid, oral cavity and larynx volumes

|  | Parapharyngeal  n (%) | IB bilateral  n (%) | II  n (%) | III  n (%) | IV  n (%) | V  n (%) |
| --- | --- | --- | --- | --- | --- | --- |
| Bilateral | 15 (48.4) | 6 (19.4) | 16 (51.6) | 16 (51.6) | 15 (48.4) | 10 (32.3) |
| Right | 5 (16.1) | 1 (3.2) | -- | -- | -- | 1 (3.2) |
| Left | -- | 1 (3.2) | -- | -- | -- | 1 (3.2) |
| No | 4 (12.9) | 6 (19.4) | -- | -- | -- | 16 (51.6) |
| Yes | 6 (19.4) | 7 (22.6) | 14 (45.2) | 14 (45.2) | 13 (41.9) | 3 (9.7) |
| Unilateral | 1 (3.2) | 10 (32.3) | 1 (3.2) | 1 (3.2) | 2 (6.5) | -- |
| Total valid | 31 | 31 | 31 | 31 | 30 | 31 |
| Missing | 0 | 0 | 0 | 0 | 1 (3.2) | 0 |

|  | Right parotid  (V26)  n (%) | Left parotid  (V26)  n (%) | Oral cavity  (V45)  n (%) | Larynx  (V45)  n (%) |
| --- | --- | --- | --- | --- |
| Yes | 31 (100) | 28 (90.3) | 30 (96.8) | 14 (45.2) |
| No | 0 (0) | 3 (9.7) | 0 (0) | 14 (45.2) |
| Total valid | 31 | 31 | 30 | 28 |
| Missing | 0 | 0 | 1 (3.2)* | 3 (9.7)* |

**Supplementary Table 2.** Systemic treatment compliance

| Cisplatin – n (% of treatment group)  0*  1  2  3 | 1 (8.3)  1 (8.3)  2 (16.7)  8 (66.7) |
| --- | --- |
| Cetuximab – n (% of treatment group)  5  6  7  8  9  12 | 3 (15.8)  1 (5.3)  1 (5.3)  7 (36.8)  6 (31.6)  1 (5.3) |

*****Patient #8 received 3 cycles of carboplatin q3w instead of cisplatin due to renal function impairment before treatment initiation.

**Supplementary Table 3**. Acute toxicity

| ACUTE TOXICITY | CRT (N=12) | | ERT (N=19) | |
| --- | --- | --- | --- | --- |
|  | Grade 1-2  n (%) | Grade 3-4  n (%) | Grade 1-2  n (%) | Grade 3-4  n (%) |
| Fatigue | 12 (100) | 0 | 15 (78.9) | 0 |
| Anorexia | 9 (75) | 0 | 9 (47.4) | 1 (5.3) |
| Dysphagia | 3 (25) | 0 | 11 (57.9) | 2 (10.5) |
| Dysphonia | 3 (25) | 0 | 0 | 0 |
| Dysgeusia | 7 (58.3) | 3 (25) | 15 (78.9) | 0 |
| Renal impairment | 2 (16.6) | 1 (8.3) | 0 | 0 |
| Oral mucositis | 8 (66.6) | 2 (16.6) | 9 (47.4) | 10 (52.6) |
| Nausea/vomits | 4 (33.3) | 1 (8.3) | 0 | 0 |
| Peripheral neuropathy | 3 (25) | 0 | 0 | 0 |
| Neutropenia | 0 | 2 (16.6) | 0 | 1 (5.3) |
| Odynophagia | 8 (66.6) | 1 (8.3) | 12 (63.2) | 0 |
| Pneumonia | 0 | 0 | 0 | 1 (5.3) |
| Radiation dermatitis | 8 (66.6) | 1 (8.3) | 14 (73.7) | 2 (10.5) |
| Skin rash | 0 | 0 | 9 (47.4) | 1 (5.3) |
| Liver toxicity | 0 | 0 | 1 (5.3) | 0 |
| Trismus | 0 | 0 | 1 (5.3) | 0 |
| Xerostomia | 12 (100) | 2 (16.6) | 4 (21.1) | 0 |
